# Investigating the Ground-State and Ionization Processes of Hydrogen Peroxide Dimers Using Sequentially Combined Theoretical Approaches

**DOI:** 10.1021/acs.jctc.5c01330

**Published:** 2025-11-24

**Authors:** José L. F. Santos, Kirk A. Peterson, Gabriel L. C. de Souza

**Affiliations:** † Instituto de Química de São Carlos, Universidade São Paulo, São Carlos, São Paulo 13566-590, Brazil; ‡ Department of Chemistry, 6760 Washington State University, Pullman, Washington 99164, United States; § Centro de Ciências da Natureza, 67828 Universidade Federal de São Carlos, Buri, São Paulo 18290-000, Brazil

## Abstract

We present a study of the structures and ionization energies (IEs) of a series of conformations of hydrogen peroxide (H_2_O_2_) dimers. Ground-state properties were computed using the coupled-cluster with single, double, and perturbative triple excitations, CCSD­(T), method with large correlation-consistent basis sets, while the ten lowest-lying IEs of the H_2_O_2_ dimer conformations were determined through the use of the equation-of-motion ionization potential coupled-cluster with single and double excitations method (EOMIP-CCSD) combined with correlation-consistent basis sets, extrapolation to the complete basis set limit, and consideration of core-correlation effects. All of the stable conformations were determined to be within 10 kJ/mol of the most stable structure (conformation I). The first IE of conformation I was determined to be 11.72 eV, while the corresponding value for conformation V was calculated as 11.58 eV. This difference (which was also noticed for other low-lying IEs and conformations) may be helpful for the assignments of experimental results. Given that the H_2_O_2_ clusters were first isolated through the use of beam experiments in the recent years, it is expected that the present work can call the attention of the community. In addition, the use of the combined theoretical approaches employed regarding the selection of the relevant conformations and the exploration of the corresponding ionization processes for the molecular clusters may present an initial guide for researchers venturing into the field.

## Graphical abstract

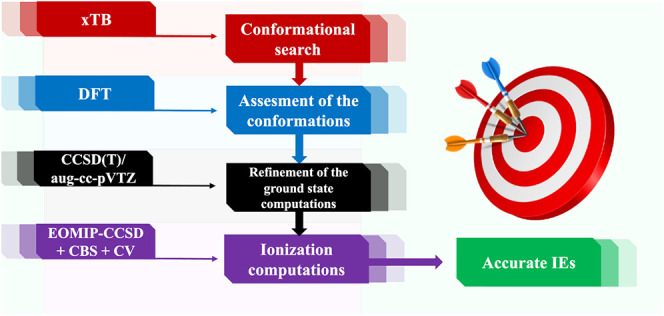

## Introduction

The aggregation of small- and medium-sized molecules (through the occurrence of different types of weakly bound forces and interactions) may be assigned as a key factor in terms of providing stability and functionality to various macromolecular systems.^1–6^ For example, Ozbagcr et al.^7^ suggested that the binding of the fast green FCF food dye with trypsin via van der Waals interactions can lead to microenvironmental and conformational changes for this particular enzyme, while other very recent works reported such interactions as contributing to the preservation of the active sites of different enzymes.^8,9^ In addition, hydrogen-bonding interactions are often associated with changes in the energetic cost of the photoexcitation processes in organic contaminants.^10–12^ These play a significant role in electron scattering cross sections and the related resonances^13^ and to realize the antioxidant power of polyphenolic species through the hydrogen-atom mechanism.^14,15^ Therefore, it is reasonable to state that understanding the impact that the referred interactions may have on the stabilities and on different quantities that are related to fundamental processes of molecular clusters can help in fingerprinting a part of the mysteries of the chemical and biological universe.

Peroxides are compounds that present the O–O bond as a signature in their chemical structures,^16^ with hydrogen peroxide (H_2_O_2_) being considered the simplest compound belonging to this class. This compound is an important biomarker that may exert some interesting roles that are associated with the defense system via oxidative biosynthesis.^17–19^ For instance, H_2_O_2_ can be generated in biological systems through oxidative burst, which involves the rapid production of a series of reactive oxygen species (ROS) in response to the attack of a given pathogen.^20^ Due to its highly oxidative power, hydrogen peroxide is considered a deleterious molecule when produced (at sufficient concentrations) in a living organism, as it can lead to cellular damage regarding either integrity or function.^21^

Beyond its ability of forming hydrogen-bonded and van der Waals complexes when interacting with chemical systems that are relevant to a variety of areas (such as atmospheric, materials, and environmental chemistry),^22–28^ when in aqueous solution, H_2_O_2_ may engage in ion–ion interactions with other ions present in water via electrostatic forces of attraction and repulsion, often facilitated by water molecules forming so-called ”water-bridged” structures.^29,30^ These interactions may be used for explaining the role of carbonate speciation on the oxidation of Fe^2+^ by H_2_O_2_, for instance.^31^ In addition, recent works suggested that H_2_O_2_ forms comparatively stronger hydrogen bonds with water via its hydrogen atoms than those formed between water molecules themselves,^32,33^ and this observation can be attributed to hydrogen peroxide functioning as a more effective hydrogen bond donor than acceptor.^34^ On the other hand, although H_2_O_2_–metal complexes play an important role in catalysis, materials science, and biotechnology, the characteristics of the interactions between hydrogen peroxide and metal cations are still unclear and widely debated. This uncertainty largely stems from the very low coordination strength of H_2_O_2_, which makes it difficult to analyze and interpret the precise nature of these interactions.^35^

From a physical-chemical standpoint, studying processes such as the detailed mechanisms of chemical reactions, electron attachment, and electron detachment happening in systems such as H_2_O_2_ may represent a difficult task. The challenges may originate due to the high instability of such species (being considerably difficult to be prepared as single molecules in gas-phase experiments), as well as due to their strong oxidizing nature.^36^ For instance, to our knowledge, one of the few experimental investigations involving dissociative electron attachment to gas-phase H_2_O_2_ was carried out by Nandi et al.^37^ In theory, employing molecular clusters could offer a promising strategy to overcome many of the experimental hurdles. However, it was only in 2020 that Pysanenko et al.^38^ made the first attempt to produce H_2_O_2_ clusters through a molecular beam, which is a considerably complicated task due to the inherent instability of hydrogen peroxide molecules. Consequently, the development and use of computational tools have become essential for exploring the stability and key processes of these species, such as ionization. These approaches can help address existing gaps in scientific understanding and aid in the future identification and characterization of various conformations of the H_2_O_2_ aggregates. To date, there are no available data in the literature regarding the existence or stability of potential conformations of H_2_O_2_ dimers. This lack of information, combined with the pioneering experimental efforts by Pysanenko et al.,^38^ served as the main motivation for conducting this current study.

In this work, we elaborated and performed a systematic investigation of the structures, energetics, and ionization energies (IEs) of a series of hydrogen peroxide clusters, more specifically, the dimers (H_2_O_2_)_2_. While stable conformations typically dominate biological interactions, high-energy forms can transiently contribute when flexibility allows necessary structural adaptations. Hence, a combination of conformational analysis and high-levels of theory, i.e., coupled-cluster, approaches was employed for exploring the forms of existence, relative stabilities, and ionization processes of a series of conformations of (H_2_O_2_)_2_. At the end, important insights were gathered regarding both the physical-chemical perspective and, secondarily, the possibility of using sequentially combined theoretical methodologies. We hope that this work may serve as motivation for computational and experimental studies of larger (H_2_O_2_)_2_ clusters for study in the near future.

## Computational Methods

### Structures and Energetics

First, the conformer-rotamer ensemble sampling tool (CREST)^39^ was used to identify low-lying conformers of the H_2_O_2_ dimers at the GFN2-xTB^40^ level of theory in the gas phase. These were subsequently fully optimized using the B3LYP exchange–correlation functional^41,42^ with the 6–311+G­(d,p) basis set,^43–46^ also in the gas phase. The Boltzmann distribution of these conformers was determined through the use of GoodVibes^47^ software. The geometry of each stable conformer found at the B3LYP/6–311+G­(d,p) level of theory was then reoptimized using the coupled-cluster with single, double, and perturbative triple excitations (CCSD­(T)) approach^48,49^ with the aug-cc-pVTZ^50,51^ basis sets (from this moment on referred to as AVTZ) in the gas phase. Harmonic vibrational frequencies were determined at this same level to characterize each structure as a minima on the potential energy surface and for evaluating zero-point vibrational corrections to the electronic energies. The density functional theory computations were accomplished with the Gaussian 09 ^53^ package, while the latest version of the MOLPRO^54^ suite of software was used for performing all of the CCSD­(T) calculations.

### Ionization Energies

The lowest-lying vertical IEs of all of the conformations of the H_2_O_2_ dimer were computed through the use of the equation-of-motion ionization potential coupled-cluster with single and double excitations approach (EOMIP-CCSD)^55^ with the aug-cc-pVnZ basis sets (*n* = D, T, Q) at the CCSD­(T)/AVTZ structures as obtained for each conformer. To explore the contributions of core–valence correlation (Δ_CV_), two EOMIP-CCSD calculations were carried out with the aug-cc-pCVnZ (*n* = D, T) basis sets (that will be denoted by ACVnZ hereafter). The first of the computations was performed considering all of the electrons correlated, while the second was accomplished through correlating only the valence electrons. Thus, the Δ_CV_ contribution was obtained by the difference between the corresponding results from these two computations. The EOMIP-CCSD total energies were extrapolated to the complete basis set (CBS) limits with the equation proposed by Martin^56^ as

1 $$E_{n}=E_{\mathrm{CBS}}+A{\left(n+1/2\right)}^{-4}$$

where *E* are the total energies and *n* is the cardinal number associated with a given basis set (for instance, *n* = 3 in the AVTZ).

The composite IEs were determined with an adapted version^57–59^ of the Feller–Peterson–Dixon (FPD) composite approach^60^ as

2 $${\mathrm{IE}}_{\mathrm{FPD}}={\mathrm{IE}}_{\mathrm{AVTZ}}+\Delta_{\mathrm{CBS}}+\Delta_{\mathrm{CV}}$$

where the Δ_CBS_ component is obtained as the difference between a given ionization energy computed at the EOMIP-CCSD/AVTZ level and its respective value estimated at the CBS limit. All of the EOMIP-CCSD computations were performed using the CFOUR^61^ package.

The sequentially combined theoretical approaches regarding the selection of the relevant conformations and the exploration of the corresponding ionization processes for molecular clusters were proposed, with their summarized scheme being shown in Figure 1.

**Figure 1:**
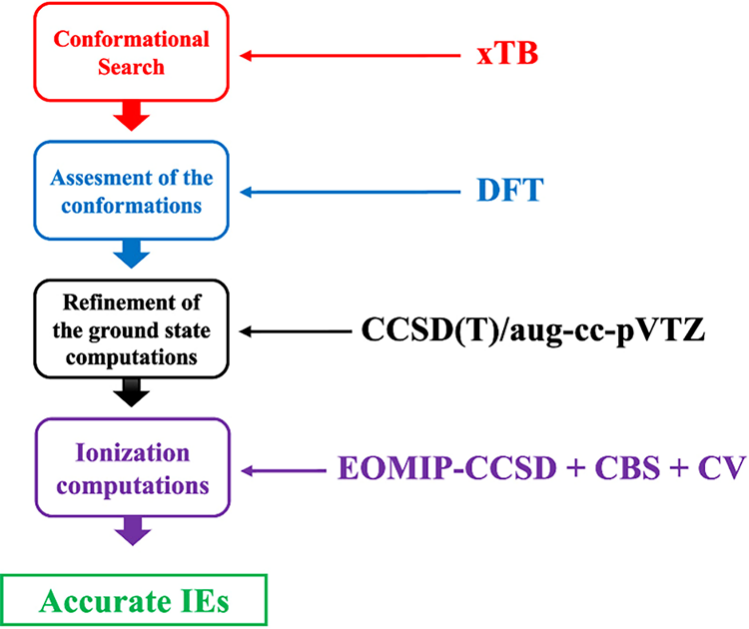
Illustration of the sequentially combined theoretical approaches employed in this work.

## Results and Discussion

Initially, a total of 13 possible rotamers of the H_2_O_2_ dimer were suggested to exist at the GFN2-xTB^40^ level of theory (in the gas phase). Subsequently, each cluster was reoptimized using the B3LYP exchange–correlation functional with the 6–311+G­(d,p) basis set; respective harmonic vibrational frequencies were computed at B3LYP/6–311+G­(d,p). After the evaluation of the vibrational frequencies of the (13) initial rotamers, seven were identified as not being minima on the corresponding potential energy surfaces. The six stable conformations of the H_2_O_2_ dimer at the B3LYP/6–311+G­(d,p) level of theory were found to have Boltzmann distributions larger than 1%. The chemical structures of the six H_2_O_2_ conformers are shown in Figure 2. Conformations I and II of the H_2_O_2_ dimer presented Boltzmann distributions of 47.50 and 30.80%, respectively, which are considerably higher than those determined for the other conformations. Conformations III and IV exhibited very close values (6.10% versus 6.20%), while conformations V and VI were found to present identical results (4.80%) for the Boltzmann distributions.

**Figure 2:**
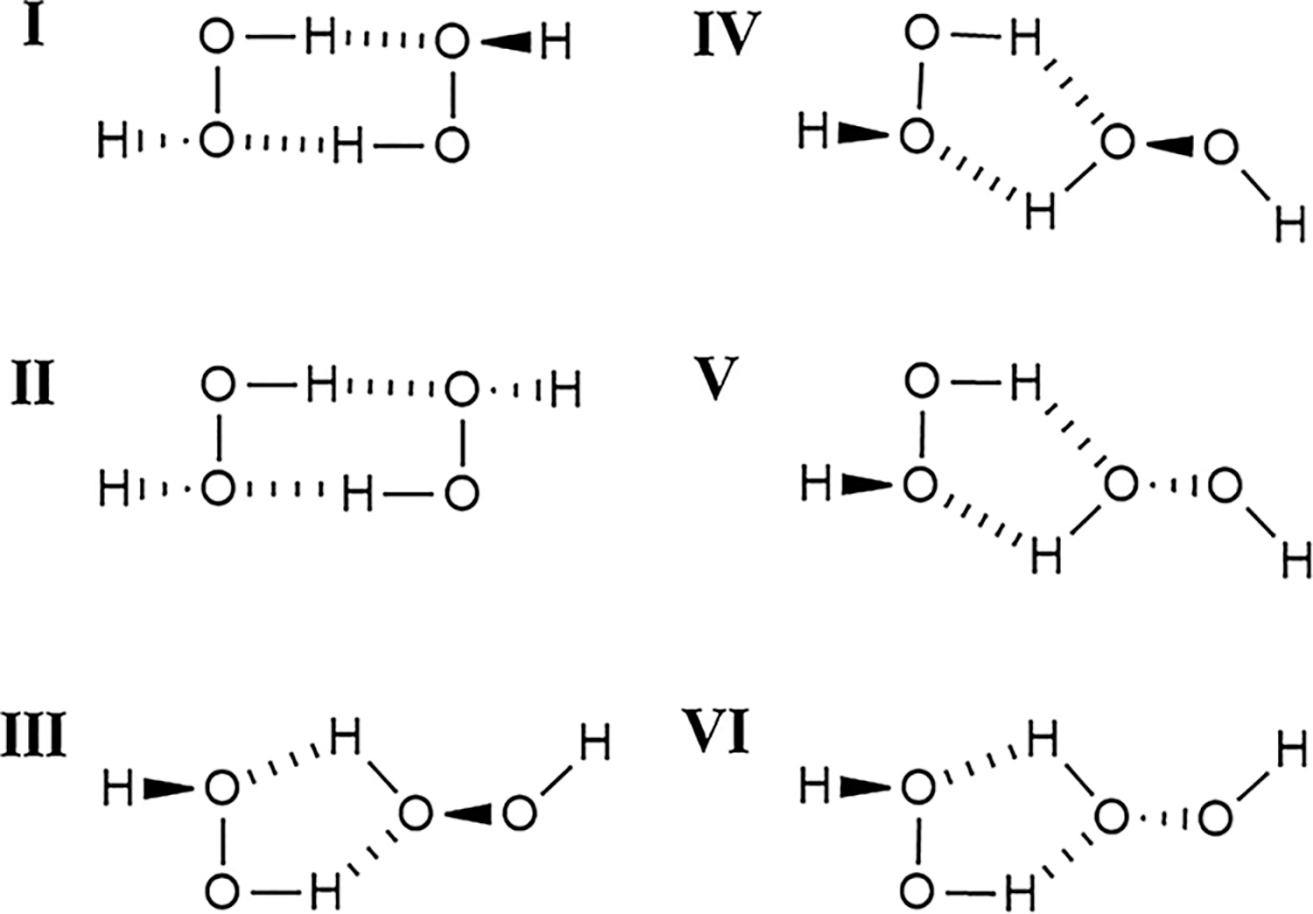
Illustration of the chemical structures of the H_2_O_2_ dimers.

The six structures of the H_2_O_2_ dimer presented in Figure 2 were used as initial guesses for optimizations at the CCSD­(T)/AVTZ level of theory. The final CCSD­(T)/AVTZ fully optimized structures with their corresponding bond distances and interactions are shown in Figure 3. In general, each system investigated presented two hydrogen bonds in its chemical structure, with conformations I and II presenting the strongest ones, as can be inferred from the O···H distances (from 1.905 to 1.907 Å). These intermolecular distances are comparable to (although slightly smaller than) the 1.9485 Å obtained by Tschumper et al.^62^ for the nonplanar open Cs conformation of the water dimer at the CCSD­(T)/TZ2P­(f,d)+diffuse level of theory, suggesting that the hydrogen-bonding interactions are marginally stronger in the H_2_O_2_ dimer in comparison to the H_2_O dimer. Overall, the intramolecular distances obtained for any of the conformations of the H_2_O_2_ dimer are comparable to the corresponding distances reported in the literature for dimers of similar molecules, such as water, ethylene glycol, and ethanol.^62–70^ Taking the conformation I (the strongest conformer) as instance, the length of the O–H bonds undergoing hydrogen interaction was determined to be 0.975 Å at the CCSD­(T)/AVTZ level of theory, which is only slightly enlarged when compared to the 0.972 and 0.965 Å reported in the case of the nonplanar open Cs conformation of the water dimer by Miliordos and Xantheas^71^ at the CCSD­(T)/AVDZ level and by Tschumper et al.^62^ at the CCSD­(T)/TZ2P­(f,d)+diffuse level, respectively. Interestingly, the structure obtained for conformation IV was found to be a mirror image when compared to that of conformation III; the same behavior was noticed in the case of conformations V and VI. These observations may be an indication that these two pairs of H_2_O_2_ dimers may represent only two actual chemical species. Cartesian coordinates of all of the optimized structures are available in the Supporting Information.

**Figure 3:**
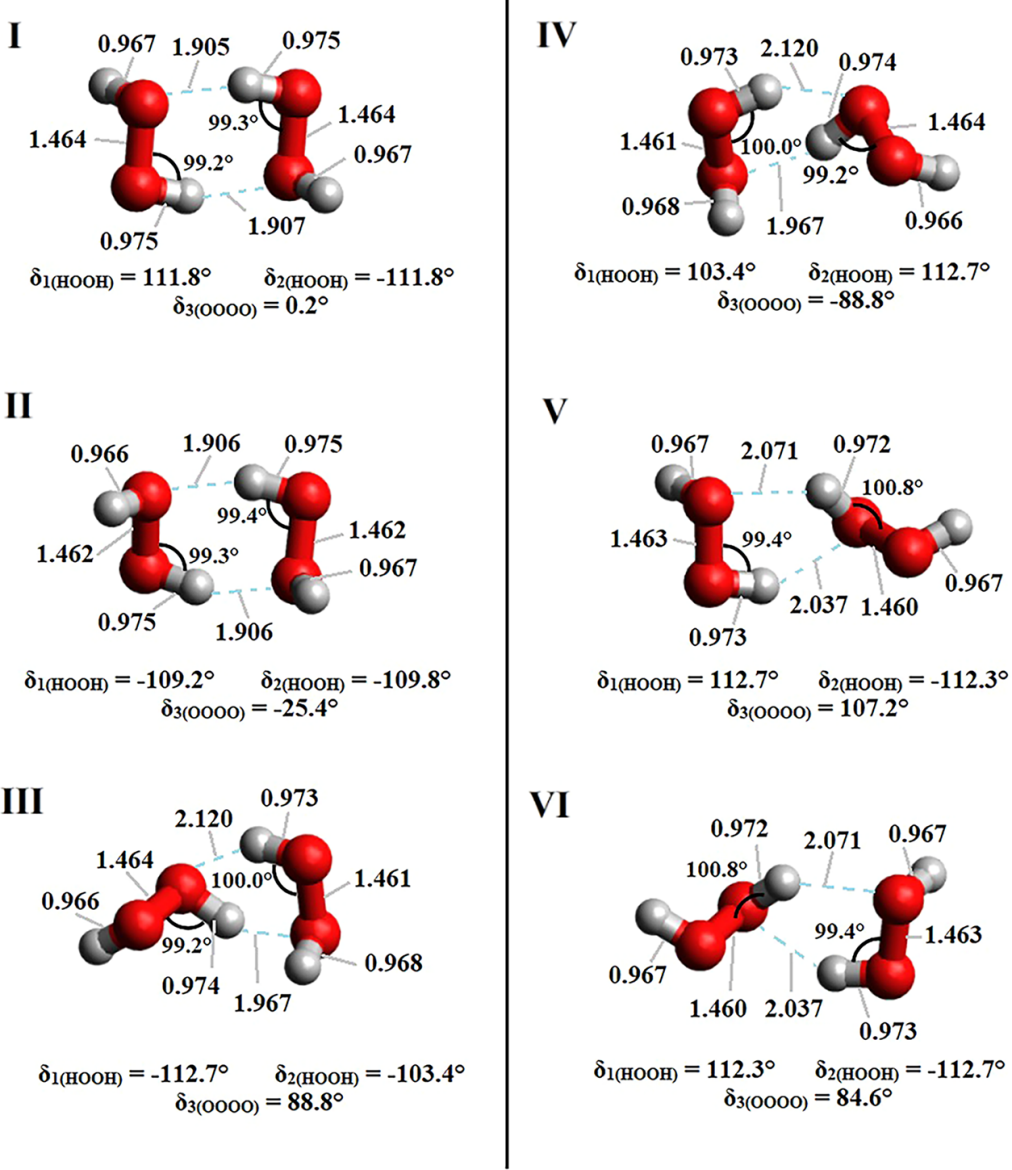
Ground-state structures of six conformations of the H_2_O_2_ dimer optimized at the CCSD­(T)/AVTZ level of theory. Inter and intramolecular bond distances are given in Å.

The relative energies (Δ*E*_conf_) and binding energies (Δ*E*_b_) determined with the CCSD­(T) approach for the conformations of the H_2_O_2_ dimers can be found in Table 1. Conformation I was found to be the strongest bound, presenting a binding energy of −37.2 kJ/mol at the CCSD­(T)/CBS­[TQ] level that decreased in magnitude to −27.4 kJ/mol at 0 K when the zero-point corrections to the electronic energies were included. The weakest bound species are the conformations V and VI with binding energies of −19.9 kJ/mol at 0K; these conformations presented exactly the same results for their energetic properties, and thus, the mirror images represent (for practical purposes) the same chemical entity. The same behavior was observed for conformations V and VI, which (along with conformations III and IV) will be grouped together and referred to as a single system from this point on. This definition is certainly an important part of the methodology, as it can yield a considerable degree of economy in terms of computational effort. The averaged binding energies obtained with CCSD­(T)/AVTZ were determined to be different by (approximately) 5% in comparison to the CCSD­(T)/CBS­[TQ] results, which suggests the use of the triple-ζ basis set as being an adequate choice to be used in these type of systems, particularly when considering the investigation of molecular clusters containing a large number of H_2_O_2_ molecules. All of the conformations of the H_2_O_2_ dimer presented binding energies that suggested these species as being more strongly bound than the H_2_O dimers. For instance, Miliordos and Xantheas^64^ estimated the Δ*E*_b_ of the H_2_O dimer as −4.99 ± 0.04 kcal/mol at the CCSD­(T)/CBS level.

**Table 1:** Relative Energies (Δ*E*_conf_) and Binding Energies (Δ*E*_b_) of the Conformations of the H_2_O_2_ Dimers, as Obtained at the CCSD­(T) Level^a^

conformation	AVTZ	CBS	ΔZPE	Δ*E*_b_^CBS^(0K)	Δ*E*_conf_^CBS^(0K)
I	–39.37	–37.20	9.79	–27.41	0.00
II	–38.46	–36.48	9.71	–26.77	0.72
III and IV	–31.40	–29.70	8.00	–21.70	7.50
V and VI	–29.11	–27.58	7.69	–19.89	9.62

a The values are given in kJ/mol.

As the chemical species under investigation in the present work consist of only hydrogen and oxygen atoms, it is expected that scalar relativity effects would play negligible roles in all their energetic properties. To verify this assumption, the binding energies of each conformation of the H_2_O_2_ dimers obtained at the CCSD­(T)/AVTZ level were compared to corresponding results determined using the aug-cc-pVTZ-DK basis sets^72^ with the second-order Douglas-Kroll-Hess scalar relativistic Hamiltonian.^73,74^ At the end, the biggest relativistic contribution was determined to be just 0.09 kJ/mol in the case of conformation II, which is consistent with the finding reported in very recent work involving H_2_CO···HNO species.^75^ Hence, taking such effects into consideration is not necessary for these actual systems and is not advised for larger clusters composed of H_2_O_2_, as the additional computations may represent a considerable increase in the computational cost (if computed as an additive contribution).

The tables containing the ten lowest-lying vertical IEs of each conformation of the H_2_O_2_ dimers, as determined using the EOMIP-CCSD approach with the AVDZ, AVTZ, and AVQZ basis sets, can be found in the Supporting Material (SM). The number of the IEs that were explored originated from the ideas prescribed by Tanaka et al.^76^ when studying the photoionization of the formaldehyde molecule. The authors concluded that a reasonable number of inner orbitals would be necessary for accurately obtaining various fundamental quantities, such as the photoionization cross sections.^76^ Hence, providing a considerably large amount of IEs may be useful as auxiliary (and input data) for other research to be based upon in the future. The IEs determined using the EOMIP-CCSD methods at the CBS limit are also available in the SM. Overall, all of the results determined for any conformation of the H_2_O_2_ dimer indicated an increase in the IEs with an increase in the size of the aug-cc-pVnZ basis set used. Taking conformation I, for example, the first IE was computed to be 11.31 eV at EOMIP-CCSD/AVDZ, 11.54 eV at EOMIP-CCSD/AVTZ, and 11.63 eV at EOMIP-CCSD/AVQZ. The CBS­[DT] IEs were found to be in considerably better agreement than CBS­[TQ] when compared with the corresponding results obtained with the AVQZ basis set. However, although this observation may be tempting for the researcher in regards of choosing CBS­[DT] over the CBS­[TQ] extrapolation, especially for large clusters containing the H_2_O_2_ molecule, the option for the CBS­[TQ] is certainly advised in every case where the quadruple-ζ computations were feasible.

The composite IEs and their individual contributions are shown in Table 2. The first IE of conformation V was determined to be 11.58 eV, which is the lowest among all of the IEs for the other conformations of the H_2_O_2_ dimer, followed by the results obtained for conformations II (11.67 eV), I (11.72 eV), and III (11.83 eV). In principle, the differences observed may be helpful for the assignments in future experiments. On the other hand, it is relevant to notice that the second IEs of conformations I and V were computed to be considerably close to the first IE of conformation III of (H_2_O_2_)_2_; more precisely, these results were determined to be −0.04 eV and +0.03 eV in comparison to 11.83 eV, respectively. This situation may bring eventual difficulties to the assignment process, given that the differences in the referred IEs are within chemical accuracy, and thus, it will demand not only the use of techniques with high spectroscopic resolution but also a careful analysis of the results measured. The core-correlation effects were found to be considerably small (with the largest contributions to the IEs being +0.03 eV), as can be noticed from the Δ_CV_ results that are also shown in Table 2. All of the raw energy values that are required for composing the final IEs are also available in the Supporting Information. In terms of the contribution of each molecular fragment to the ionization of the dimers, it is possible to notice from the highest occupied molecular orbital (HOMO) to the HOMO −6 plots (also available in the Supporting Information) that the ionization processes have a delocalized nature in all of the conformations of the (H_2_O_2_)_2_.

**Table 2:** Composite EOMIP-CCSD Vertical IEs (in eVs) of the H_2_O_2_ Dimers

conformation	I	II	III = IV	V = VI
	IE 1			
*E*_AVTZ_	11.54	11.50	11.65	11.41
Δ_CBS[TQ]_	0.15	0.14	0.15	0.14
Δ_CV_	0.03	0.03	0.03	0.03
IP_FPD_	11.72	11.67	11.83	11.58
	IE 2			
*E*_AVTZ_	11.61	11.75	11.74	11.69
Δ_CBS[TQ]_	0.15	0.14	0.14	0.14
Δ_CV_	0.03	0.03	0.03	0.03
IP_FPD_	11.79	11.92	11.91	11.86
	IE 3			
*E*_AVTZ_	12.27	12.30	12.44	12.56
Δ_CBS[TQ]_	0.14	0.14	0.14	0.14
Δ_CV_	0.03	0.03	0.03	0.03
IP_FPD_	12.44	12.47	12.61	12.73
	IE 4			
*E*_AVTZ_	13.37	13.10	12.71	12.71
Δ_CBS[TQ]_	0.14	0.14	0.14	0.14
Δ_CV_	0.03	0.03	0.03	0.03
IP_FPD_	13.54	13.27	12.88	12.88
	IE 5			
*E*_AVTZ_	15.48	15.47	15.42	15.22
Δ_CBS[TQ]_	0.12	0.13	0.12	0.13
Δ_CV_	0.02	0.02	0.02	0.03
IP_FPD_	15.62	15.62	15.56	15.38
	IE 6			
*E*_AVTZ_	15.51	15.60	15.62	15.64
Δ_CBS[TQ]_	0.12	0.12	0.12	0.12
Δ_CV_	0.03	0.03	0.02	0.02
IP_FPD_	15.66	15.75	15.76	15.78
	IE 7			
*E*_AVTZ_	16.74	16.60	16.95	17.06
Δ_CBS[TQ]_	0.13	0.13	0.13	0.12
Δ_CV_	0.02	0.02	0.02	0.02
IP_FPD_	16.89	16.75	17.10	17.20
	IE 8			
*E*_AVTZ_	18.19	18.26	17.90	17.81
Δ_CBS[TQ]_	0.12	0.12	0.12	0.11
Δ_CV_	0.02	0.02	0.02	0.02
IP_FPD_	18.33	18.40	18.04	17.94
	IE 9			
*E*_AVTZ_	18.64	18.70	18.61	18.43
Δ_CBS[TQ]_	0.11	0.11	0.11	0.11
Δ_CV_	0.02	0.02	0.02	0.02
IP_FPD_	18.77	18.83	18.74	18.56
	IE 10			
*E*_AVTZ_	18.74	18.72	18.73	18.75
Δ_CBS[TQ]_	0.11	0.11	0.11	0.10
Δ_CV_	0.02	0.02	0.02	0.02
IP_FPD_	18.87	18.85	18.86	18.87

The composite (vertical) IEs and their individual contributions for the H_2_O_2_ monomer are shown in Table 3. Overall, the Δ_CBS[TQ]_ and Δ_CV_ contributions to the five lowest-lying IEs of the H_2_O_2_ molecule were found to be comparable to those obtained for all of the IEs of the dimers. However, a pronounced increase in these contributions was noticed in the case of the high-lying IEs. The first composite vertical ionization energy of H_2_O_2_ was computed to be 11.71 eV, a value that is approximately 1 eV larger than the 10.638 ± 0.012 eV *adiabatic* ionization energy as obtained by Changala et al.^77^ with an approach based on high-level computations, a difference that is due primarily to the changes in the molecular structure after the ionization, with the ionized geometry presenting a dihedral of 180.0° (see Figure 4). In the current work, the CCSD­(T)/AVTZ vertical and equilibrium IEs are 11.67 and 10.57 eV, respectively. Interestingly, the first vertical IE of the H_2_O_2_ monomer was determined to be very close to the corresponding result obtained for conformation I of the H_2_O_2_ dimer. Actually, the molecular system presented the vertical IE_1_ as being only 0.01 eV lower than the respective ionization energy of conformation I of the H_2_O_2_ dimer. This observation could suggest that knowing only the IEs of the isolated molecule might (perhaps) suffice for describing the ionization processes of the most stable conformation of the H_2_O_2_ dimer, which can actually be misleading, as the behavior vanishes when comparing the other IEs, with the differences between the results determined for the monomer and dimers continuing to grow especially for the high-lying IEs. In terms of comparison with results obtained experimentally for a similar system, the first and second IEs obtained for any conformation of the H_2_O_2_ dimer are slightly lower in comparison to the experimental results reported for the water dimer by Tomoda et al.^78^ through the use of a nozzle beam photoelectron spectrometer; the authors obtained these IEs as being 12.1 ± 0.1 eV and 13.2 ± 0.2 eV, respectively.

**Figure 4:**
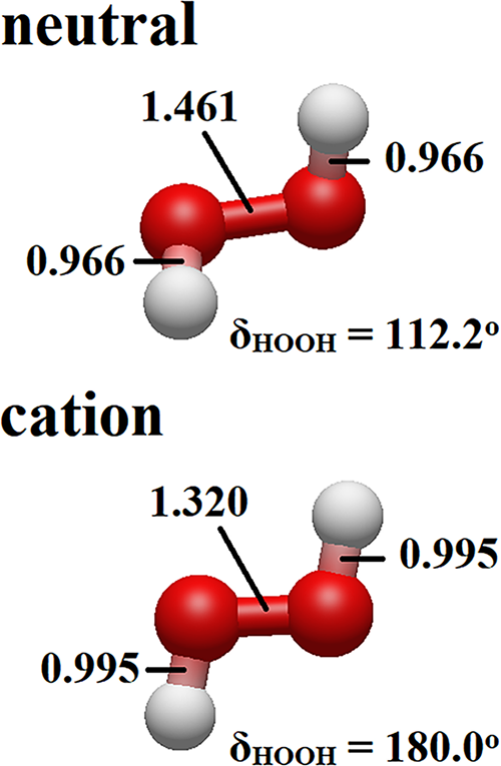
Structures of the neutral and cationic forms of the H_2_O_2_ monomer, as optimized at the CCSD­(T)/AVTZ level of theory. Inter- and intramolecular bond distances are given in Å.

**Table 3:** Composite EOMIP-CCSD Vertical IEs (in eVs) of the H_2_O_2_ Molecule

IE	*E*_AVTZ_	Δ_CBS[TQ]_	Δ_CV_	IP_FPD_
1	11.54	0.14	0.03	11.71
2	12.58	0.13	0.03	12.74
3	15.38	0.13	0.02	15.53
4	17.40	0.12	0.02	17.54
5	18.55	0.10	0.02	18.67
6	21.37	0.31	0.15	21.83
7	23.02	0.31	0.15	23.48
8	23.78	0.29	0.15	24.22
9	24.28	0.29	0.14	24.71
10	26.32	0.29	0.14	26.75

In a manner similar to that observed in the comparison of the vertical and the adiabatic IEs obtained for the H_2_O_2_ monomer, the latter determined for the H_2_O_2_ dimers were also found to be considerably lower than the corresponding vertical IEs. In particular, the first equilibrium IEs for dimers I, II, III–IV, and V–VI are (in eV) 9.52, 9.51, 9.44, and 9.41, respectively, as calculated at the CCSD­(T)/AVTZ level of theory. The corresponding CCSD­(T)/AVTZ values for the vertical IE are 11.70, 11.70, 11.78, and 11.56 eV, respectively. Interestingly, the order of these IEs is also slightly different from that noticed when not considering the reorganization of the molecular geometry after the ionization. In comparison to the EOMIP-CCSD vertical IEs of Table 2, the CCSD­(T) values are about 0.15 to 0.20 eV larger. This is only slightly larger than the difference between EOMIP-CCSD and CCSD­(T) for the monomer. Pronounced changes in the chemical structures of the cations of the H_2_O_2_ dimers were also seen, with each of the two monomers being strongly bonded by the plane-oriented O···H···O interaction. The structures of the cationic forms of the H_2_O_2_ dimer, as optimized at the R/UCCSD­(T)/aVTZ level of theory (restricted open-shell Hartree–Fock orbitals with unrestricted CCSD as implemented in Molpro^79^), can be found in Figure 5. The large structural changes upon ionization lead to differences between the vertical and equilibrium IEs of about a factor of 2 larger than what is calculated for the monomer, i.e., about −2.2 eV in the dimers compared to −1.0 eV in the monomer.

**Figure 5:**
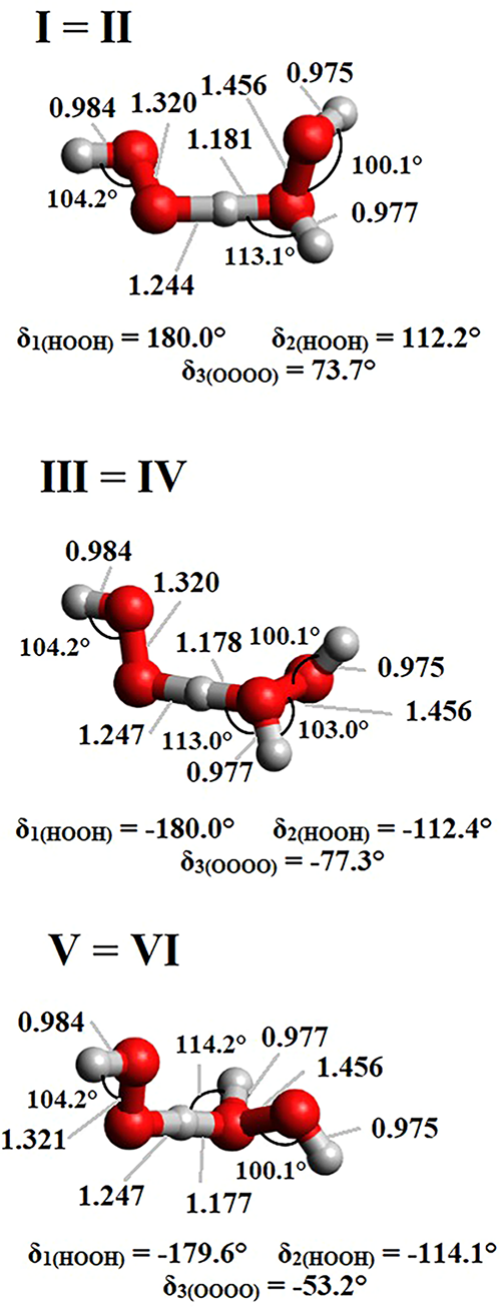
Structures of the cationic forms of the H_2_O_2_ dimer as optimized at the CCSD­(T)/aVTZ level of theory. Inter- and intramolecular bond distances are given in Å.

## Conclusions

We performed an investigation of the ground-state binding energies and ionization processes of a series of hydrogen peroxide clusters. The most stable aggregation form of the H_2_O_2_ dimer was found to be conformation I, which was determined as being only 0.72 kJ/mol lower than conformation II at the CCSD­(T)/CBS level of theory; the other stable conformations were calculated to be within 10 kJ/mol from conformation I at the same CCSD­(T)/CBS level of theory. The differences observed in the IEs of (H_2_O_2_)_2_ can be important for the assignments of experimental data regarding the identification of the different conformations of such clusters. Given that the H_2_O_2_ clusters were first isolated through beam experiments in recent years, it is expected that the present work might call the attention of the scientific community to this system, and these results will hopefully motivate future experimental and theoretical investigations. More specifically, investigations targeting the exploration of how the different H_2_O_2_ dimer conformers would behave when interacting with macromolecular, biological, and food systems would certainly be welcome.

## Supplementary Material



## Notes

Individual energy contributions to the vertical ionization energies for all of the conformations of the H_2_O_2_ dimer, raw electronic energy values, Cartesian coordinates of the optimized structures, and molecular orbital plots (PDF)
