# Supplementary material for: Investigating the Ground-State and Ionization Processes of Hydrogen Peroxide Dimers Using Sequentially Combined Theoretical Approaches
Source: J Chem Theory Comput. 2025 Nov 24;21(23):11969–77. doi: 10.1021/acs.jctc.5c01330 (PMC12874355; doi:10.1021/acs.jctc.5c01330)
Supplement: Supplementary file 1 [file ct5c01330_si_001.pdf]

Supporting Information: Investigating the ground-state and ionization processes of hydrogen peroxide dimers using sequentially combined theoretical approaches

José L. F. Santos,[†] Kirk A. Peterson,[*,‡] and Gabriel L. C. de Souza[*,¶]

†*Instituto de Química de São Carlos, Universidade São Paulo, São Carlos, São Paulo, 13566-590 Brazil*

‡*Department of Chemistry, Washington State University, Pullman, Washington 99164 USA*

¶*Centro de Ciências da Natureza, Universidade Federal de São Carlos, Buri, São Paulo, 18290-000 Brazil*

E-mail: kipeters@wsu.chem.edu; gabriellcs@ufscar.br

Phone: +1-509-335-7867; +55-15-3256-9000

Table S1: Vertical ionization energies (IEs) for the conformation I of the H_2O_2 dimer as determined using the frozen-core EOMIP-CCSD/aug-cc-pVnZ (n = D, T, and Q) approaches along with CBS limit extrapolations. All the values are given in eV.

IE	$\mathbf{E}_{\text{AVDZ}}$	$\mathbf{E}_{\text{AVTZ}}$	$\mathbf{E}_{\text{AVQZ}}$	$\mathbf{E}_{\text{CBS[DT]}}$	$\mathbf{E}_{\text{CBS[TQ]}}$
1	11.31	11.54	11.63	11.62	11.69
2	11.37	11.61	11.70	11.70	11.76
3	12.05	12.27	12.36	12.35	12.41
4	13.16	13.37	13.46	13.45	13.51
5	15.32	15.48	15.56	15.53	15.60
6	15.34	15.51	15.59	15.57	15.63
7	16.58	16.74	16.82	16.80	16.87
8	18.05	18.19	18.26	18.24	18.31
9	18.54	18.64	18.71	18.67	18.75
10	18.64	18.74	18.81	18.78	18.85

Table S2: Core correlation effects for the vertical ionization energies (IEs) for the conformation I of the H_2O_2 dimer as determined using EOMIP-CCSD/aug-cc-pCVnZ (n = D and T) approaches. The values are given in eV.

IE	$\mathbf{E}^{\text{FC}}_{\text{ACVDZ}}$	$\mathbf{E}^{\text{FC}}_{\text{ACVTZ}}$	$\Delta_{\text{CV}}\mathbf{(DZ)}$	$\Delta_{\text{CV}}\mathbf{(TZ)}$
1	11.32	11.56	0.008	0.03
2	11.38	11.63	0.008	0.03
3	12.06	12.29	0.008	0.03
4	13.17	13.38	0.008	0.03
5	15.33	15.49	0.01	0.02
6	15.35	15.52	0.01	0.03
7	16.60	16.75	0.01	0.02
8	18.06	18.20	0.01	0.02
9	18.55	18.64	0.01	0.02
10	18.65	18.75	0.01	0.02

Table S3: Raw values used for computing the vertical ionization energies (IEs) for the conformation I of the H_2O_2 dimer as determined using the frozen-core EOMIP-CCSD/aug-cc-pVnZ (n = D, T, and Q) approaches. The values are given in hartrees.

**State**	$\mathbf{E}_{AVDZ}$	$\mathbf{E}_{AVTZ}$	$\mathbf{E}_{AVQZ}$
Ground	-302.47404008086778	-302.72151791662054	-302.79771503118968
1	-302.05841524219119	-302.29733224620679	-302.37015656031366
2	-302.05606290647114	-302.29480205835358	-302.36763371700397
3	-302.03110849075875	-302.27050513077216	-302.34340387687075
4	-301.99027906784283	-302.23011419395255	-302.30319275451569
5	-301.91108551055800	-302.15274347738722	-302.22602341749064
6	-301.91028387017531	-302.15164546378423	-302.22487115378965
7	-301.86466234487330	-302.10632311655110	-302.17959147261763
8	-301.81078028031720	-302.05307334030812	-302.12657759896200
9	-301.79266447816065	-302.03661186734570	-302.11024764915339
10	-301.78895136595548	-302.03271480162562	-302.10635329399366

Table S4: Raw values used for computing the vertical ionization energies (IEs) for the conformation I of the H_2O_2 dimer as determined using the EOMIP-CCSD/aug-cc-pCVnZ (n = D and T) approach with all electrons correlated. The values are given in hartrees.

**State**	$\mathbf{E}_{ACVDZ}$	$\mathbf{E}_{ACVTZ}$
Ground	-302.63804053463946	-302.94713544259230
1	-302.22175269674989	-302.52119441909537
2	-302.21939875034911	-302.51866529864412
3	-302.19444006723148	-302.49445075323973
4	-302.15364025361453	-302.45418774973064
5	-302.07442277801289	-302.37701196294273
6	-302.07360630668597	-302.37588187905538
7	-302.02794611644100	-302.33065167697214
8	-301.97410622257871	-302.27754707230395
9	-301.95617508209722	-302.26117613615685
10	-301.95244859277159	-302.25725839560215

Table S5: Raw values used for computing the vertical ionization energies (IEs) for the conformation I of the H_2O_2 dimer as determined using the frozen-core EOMIP-CCSD/aug-cc-pCVnZ (n = D and T) approaches. The values are given in hartrees.

**State**	$\mathbf{E}^{FC}_{ACVDZ}$	$\mathbf{E}^{FC}_{ACVTZ}$
Ground	-302.48552675666889	-302.73320751466423
1	-302.06954487544289	-302.30849887368726
2	-302.06719026596232	-302.30595513988669
3	-302.04222199749853	-302.28168963630526
4	-302.00141506555798	-302.24134018151915
5	-301.92214817790625	-302.16399114164909
6	-301.92134406902471	-302.16288265773164
7	-301.87567765680927	-302.11758700672789
8	-301.82182393270364	-302.06439108204927
9	-301.80388838683876	-302.04806298073692
10	-301.80017475190203	-302.04415162805776

Table S6: Cartesian coordinates of the conformation I of the H_2O_2 dimer as optimized at the CCSD(T)/aug-cc-pVTZ level of theory in the gas-phase. Values are given in Å

Atom	X	Y	Z
O	1.4699449294	0.7535129510	0.0143346538
O	1.3567781200	-0.7025695461	0.1139808231
O	-1.3588137486	0.7016535604	-0.1161263356
O	-1.4714028534	-0.7541269046	-0.0101337673
H	1.7934521680	-0.9629203001	-0.7085773199
H	0.5283701706	0.9756140302	-0.1093572143
H	-1.7893383286	0.9650818076	0.7087114945
H	-0.5289904574	-0.9762455983	0.1071676657

Table S7: Cartesian coordinates of the cation of conformation I of the H_2O_2 dimer as optimized at the CCSD(T)/aug-cc-pVTZ level of theory in the gas-phase. Values are given in Å

Atom	X	Y	Z
O	1.5987248426	0.5399307034	0.2968528134
O	0.9287143627	-0.4053180257	-0.5857365969
O	-1.8384171063	0.5413772542	0.1695453294
O	-1.2471108146	-0.6013675767	0.4649601731
H	1.4830257329	-1.2064325944	-0.5119657702
H	1.9259912732	1.1836566506	-0.3580137231
H	-2.7217717429	0.4736081464	0.5987514777
H	-0.1291565475	-0.5254545579	-0.0743937035

Table S8: Vertical ionization energies (IEs) for the conformation II of the H_2O_2 dimer as determined using the frozen-core EOMIP-CCSD/aug-cc-pVnZ (n = D, T, and Q) approaches along with CBS limit extrapolations. All the values are given in eV.

**IE**	$\mathbf{E}_{\mathrm{AVDZ}}$	$\mathbf{E}_{\mathrm{AVTZ}}$	$\mathbf{E}_{\mathrm{AVQZ}}$	$\mathbf{E}_{\mathrm{CBS[DT]}}$	$\mathbf{E}_{\mathrm{CBS[TQ]}}$
1	11.26	11.50	11.59	11.58	11.64
2	11.52	11.75	11.84	11.83	11.89
3	12.09	12.30	12.39	12.38	12.44
4	12.88	13.10	13.19	13.18	13.24
5	15.32	15.47	15.55	15.53	15.60
6	15.43	15.60	15.68	15.66	15.72
7	16.44	16.60	16.68	16.66	16.73
8	18.12	18.26	18.34	18.31	18.38
9	18.60	18.70	18.77	18.74	18.81
10	18.62	18.72	18.79	18.75	18.83

Table S9: Core correlation effects for the vertical ionization energies (IEs) for the conformation II of the H_2O_2 dimer as determined using EOMIP-CCSD/aug-cc-pCVnZ (n = D and T) approaches. The values are given in eV.

IE	$\mathbf{E}^{\mathrm{FC}}_{\mathrm{ACVDZ}}$	$\mathbf{E}^{\mathrm{FC}}_{\mathrm{ACVTZ}}$	Δ_{CV}(DZ)	Δ_{CV}(TZ)
1	11.27	11.51	0.008	0.03
2	11.53	11.77	0.008	0.03
3	12.10	12.32	0.008	0.03
4	12.89	13.11	0.008	0.03
5	15.33	15.48	0.01	0.02
6	15.44	15.61	0.01	0.03
7	16.45	16.61	0.01	0.02
8	18.14	18.28	0.01	0.02
9	18.61	18.71	0.01	0.02
10	18.63	18.72	0.01	0.02

Table S10: Raw values used for computing the vertical ionization energies (IEs) for the conformation II of the H_2O_2 dimer as determined using the frozen-core EOMIP-CCSD/aug-cc-pVnZ (n = D, T, and Q) approaches. The values are given in hartrees.

State	$\mathbf{E}_{AVDZ}$	$\mathbf{E}_{AVTZ}$	$\mathbf{E}_{AVQZ}$
Ground	-302.47365389143391	-302.72126297596384	-302.79751227420905
1	-302.05978737469650	-302.29871784395260	-302.37157999335875
2	-302.05043637091580	-302.28942374335668	-302.36234774251972
3	-302.02932388731546	-302.26911017996667	-302.34210860547063
4	-302.00019779498541	-302.23986247225400	-302.31291928168287
5	-301.91074199461502	-302.15266517428472	-302.22602050902537
6	-301.90657336184654	-302.14810117078059	-302.22140140048441
7	-301.86946301393999	-302.11115799736331	-302.18445176576006
8	-301.80766718211373	-302.05005598470098	-302.12361851571785
9	-301.79010887358055	-302.03404258360331	-302.10773542872062
10	-301.78921654999260	-302.03338535885865	-302.10708898116366

Table S11: Raw values used for computing the vertical ionization energies (IEs) for the conformation II of the H_2O_2 dimer as determined using the EOMIP-CCSD/aug-cc-pCVnZ (n = D and T) approach with all electrons correlated. The values are given in hartrees.

**State**	$\mathbf{E}_{ACVDZ}$	$\mathbf{E}_{ACVTZ}$
Ground	-302.63764133814931	-302.94689827260061
1	-302.22310944434508	-302.52258858003910
2	-302.21376986282525	-302.51333684237187
3	-302.19265103099963	-302.49310737479607
4	-302.16353660394793	-302.46390014107106
5	-302.07406692277141	-302.37696752875098
6	-302.06989350804821	-302.37237118997018
7	-302.03272720508238	-302.33548731713222
8	-301.97097348863849	-302.27455003930521
9	-301.95359849417758	-302.25860810314509
10	-301.95272030491833	-302.25797622716698

Table S12: Raw values used for computing the vertical ionization energies (IEs) for the conformation II of the H_2O_2 dimer as determined using the frozen-core EOMIP-CCSD/aug-cc-pCVnZ (n = D and T) approaches. The values are given in hartrees.

**State**	$\mathbf{E}^{FC}_{ACVDZ}$	$\mathbf{E}^{FC}_{ACVTZ}$
Ground	-302.48513670087738	-302.73296572855725
1	-302.07091090354851	-302.30988893570731
2	-302.06156615064629	-302.30059897939179
3	-302.04043337138614	-302.28031793276733
4	-302.01132922844255	-302.25108517854841
5	-301.92179348998741	-302.16392530332939
6	-301.91764027358084	-302.15936013974488
7	-301.88047129739442	-302.12242931393672
8	-301.81870099540441	-302.06138253521493
9	-301.80133168629516	-302.04549559665634
10	-301.80044172762422	-302.04485390436173

Table S13: Cartesian coordinates of the conformation II of the H_2O_2 dimer as optimized at the CCSD(T)/aug-cc-pVTZ level of theory in the gas-phase. Values are given in Å

Atom	X	Y	Z
O	1.4662315045	0.7135640696	-0.4003652869
O	1.3409792674	-0.7074718640	-0.0795368272
O	-1.3387051998	0.7072732028	-0.0782387294
O	-1.4647141838	-0.7133123932	-0.4012551605
H	1.7792343246	-0.7318453844	0.7815445128
H	0.5341419803	0.9814835463	-0.2991138543
H	-0.5325470378	-0.9816757937	-0.3018812134
H	-1.7846206553	0.7319846166	0.7788465588

Table S14: Cartesian coordinates of the cation of conformation II of the H_2O_2 dimer as optimized at the CCSD(T)/aug-cc-pVTZ level of theory in the gas-phase. Values are given in Å

Atom	X	Y	Z
O	1.5521844730	0.7168264266	0.2084115324
O	0.9884427242	-0.4927640611	-0.3748614002
O	-1.8829732789	0.4127779932	-0.1326894464
O	-1.2409501514	-0.4972992606	0.5761134866
H	1.5720460897	-1.1962492093	-0.0298440728
H	1.8935906660	1.1515089611	-0.5946249316
H	-0.0943083989	-0.5201492898	0.0931517296
H	-2.7880321237	0.4253484399	0.2543431024

Table S15: Vertical ionization energies (IEs) for the conformation III of the H_2O_2 dimer as determined using the frozen-core EOMIP-CCSD/aug-cc-pVnZ (n = D, T, and Q) approaches along with CBS limit extrapolations. All the values are given in eV.

IE	$\mathbf{E}_{\mathrm{AVDZ}}$	$\mathbf{E}_{\mathrm{AVTZ}}$	$\mathbf{E}_{\mathrm{AVQZ}}$	$\mathbf{E}_{\mathrm{CBS[DT]}}$	$\mathbf{E}_{\mathrm{CBS[TQ]}}$
1	11.42	11.65	11.74	11.73	11.80
2	11.51	11.74	11.83	11.82	11.88
3	12.23	12.44	12.53	12.52	12.58
4	12.49	12.71	12.79	12.78	12.85
5	15.25	15.42	15.50	15.47	15.54
6	15.46	15.62	15.70	15.67	15.74
7	16.79	16.95	17.03	17.01	17.08
8	17.75	17.90	17.98	17.96	18.02
9	18.52	18.61	18.68	18.64	18.72
10	18.64	18.73	18.80	18.76	18.84

Table S16: Core correlation effects for the vertical ionization energies (IEs) for the conformation III of the H_2O_2 dimer as determined using EOMIP-CCSD/aug-cc-pCVnZ (n = D and T) approaches. The values are given in eV.

IE	$\mathbf{E}^{\mathrm{FC}}_{\mathrm{ACVDZ}}$	$\mathbf{E}^{\mathrm{FC}}_{\mathrm{ACVTZ}}$	$\Delta_{\mathrm{CV}}(\mathbf{DZ})$	$\Delta_{\mathrm{CV}}(\mathbf{TZ})$
1	11.43	11.67	0.008	0.03
2	11.51	11.75	0.008	0.03
3	12.24	12.46	0.008	0.03
4	12.50	12.72	0.008	0.03
5	15.27	15.43	0.01	0.02
6	15.48	15.63	0.01	0.02
7	16.80	16.96	0.01	0.02
8	17.76	17.92	0.01	0.02
9	18.52	18.62	0.01	0.02
10	18.64	18.74	0.01	0.02

Table S17: Raw values used for computing the vertical ionization energies (IEs) for the conformation III of the H_2O_2 dimer as determined using the frozen-core EOMIP-CCSD/aug-cc-pVnZ (n = D, T, and Q) approaches. The values are given in hartrees.

**State**	$\mathbf{E}_{AVDZ}$	$\mathbf{E}_{AVTZ}$	$\mathbf{E}_{AVQZ}$
Ground	-302.47152500047196	-302.71870126228447	-302.79499630731016
1	-302.05195170347525	-302.29053514160557	-302.36346180601106
2	-302.04872719483774	-302.28733163561770	-302.36030257489256
3	-302.02224139474015	-302.26146270024179	-302.33453401332895
4	-302.01243726119071	-302.25175153301262	-302.32480455353215
5	-301.91093817286122	-302.15221471173669	-302.22555609724242
6	-301.90321478531320	-302.14469798642642	-302.21808781636253
7	-301.85450561406526	-302.09571792381297	-302.16914175756779
8	-301.81920404897539	-302.06073359424522	-302.13427405757415
9	-301.79102703844006	-302.03478150483102	-302.10852060413754
10	-301.78662435094293	-302.03037652217813	-302.10412849995919

Table S18: Raw values used for computing the vertical ionization energies (IEs) for the conformation III of the H_2O_2 dimer as determined using the EOMIP-CCSD/aug-cc-pCVnZ (n = D and T) approach with all electrons correlated. The values are given in hartrees.

**State**	$\mathbf{E}_{ACVDZ}$	$\mathbf{E}_{ACVTZ}$
Ground	-302.63546396259960	-302.94433384141962
1	-302.21523327118655	-302.51442430685751
2	-302.21202253296417	-302.51125074602896
3	-302.18553524985896	-302.48546865010326
4	-302.17572552848901	-302.47576150236875
5	-302.07421104097369	-302.37648661350215
6	-302.06650857130779	-302.36900035148705
7	-302.01773772107197	-302.32008673758185
8	-301.98243224869213	-302.28517519568391
9	-301.95449656492798	-302.25936685566268
10	-301.95008860567248	-302.25496980549616

Table S19: Raw values used for computing the vertical ionization energies (IEs) for the conformation III of the H_2O_2 dimer as determined using the frozen-core EOMIP-CCSD/aug-cc-pCVnZ (n = D and T) approaches. The values are given in hartrees.

**State**	$\mathbf{E}^{FC}_{ACVDZ}$	$\mathbf{E}^{FC}_{ACVTZ}$
Ground	-302.48301771826010	-302.73043763145205
1	-302.06309064808607	-302.30174987467666
2	-302.05987431963888	-302.29854015993646
3	-302.03337918983738	-302.27270792977959
4	-302.02356725669779	-302.26299264886768
5	-301.92200591173156	-302.16350016695526
6	-301.91429999893245	-302.15600449816543
7	-301.86553601525577	-302.10702753282555
8	-301.83022422973124	-302.07205668402850
9	-301.80227686185276	-302.04628767109335
10	-301.79786824348139	-302.04188178799325

Table S20: Cartesian coordinates of the conformation III of the H_2O_2 dimer as optimized at the CCSD(T)/aug-cc-pVTZ level of theory in the gas-phase. Values are given in Å

Atom	X	Y	Z
O	-1.5002722405	0.1078974676	-0.5674586875
O	-1.0570708835	-0.5079019003	0.6844094267
O	1.6494195142	-0.7299054845	-0.3410684346
O	1.4657891989	0.5605230353	0.3178358449
H	-0.3523127855	0.1204439466	0.9257218463
H	-2.3848413206	0.3965600778	-0.3063962073
H	1.4295558755	1.1431522174	-0.4537898177
H	0.7497326414	-1.0907693600	-0.2592539708

Table S21: Cartesian coordinates of the cation of conformation III of the H_2O_2 dimer as optimized at the CCSD(T)/aug-cc-pVTZ level of theory in the gas-phase. Values are given in Å

Atom	X	Y	Z
O	-1.9228081755	-0.1001022707	-0.1951899355
O	-1.1638725240	0.6544947671	0.5779345911
O	1.4170670512	-0.9621654132	0.2025092712
O	1.0638283381	0.3475950526	-0.3275917367
H	-0.0129764166	0.5198934529	0.1178252017
H	-2.8285979673	0.0112714485	0.1737952275
H	1.7387353610	0.9340267716	0.0663301375
H	1.7086243332	-1.4050138088	-0.6156127567

Table S22: Vertical ionization energies (IEs) for the conformation V of the H_2O_2 dimer as determined using the frozen-core EOMIP-CCSD/aug-cc-pVnZ (n = D, T, and Q) approaches along with CBS limit extrapolations. All the values are given in eV.

**IE**	$\mathbf{E}_{\mathrm{AVDZ}}$	$\mathbf{E}_{\mathrm{AVTZ}}$	$\mathbf{E}_{\mathrm{AVQZ}}$	$\mathbf{E}_{\mathrm{CBS[DT]}}$	$\mathbf{E}_{\mathrm{CBS[TQ]}}$
1	11.18	11.41	11.50	11.49	11.55
2	11.45	11.69	11.78	11.77	11.83
3	12.35	12.56	12.65	12.63	12.70
4	12.50	12.71	12.80	12.79	12.85
5	15.06	15.22	15.30	15.28	15.35
6	15.48	15.64	15.72	15.69	15.76
7	16.90	17.06	17.13	17.11	17.18
8	17.65	17.81	17.88	17.86	17.92
9	18.35	18.43	18.50	18.46	18.54
10	18.65	18.75	18.81	18.78	18.85

Table S23: Core correlation effects for the vertical ionization energies (IEs) for the conformation V of the H_2O_2 dimer as determined using EOMIP-CCSD/aug-cc-pCVnZ (n = D and T) approaches. The values are given in eV.

**IE**	$\mathbf{E}^{\mathrm{FC}}_{\mathrm{ACVDZ}}$	$\mathbf{E}^{\mathrm{FC}}_{\mathrm{ACVTZ}}$	Δ_{CV}**(DZ)**	Δ_{CV}**(TZ)**
1	11.18	11.42	0.008	0.03
2	11.46	11.70	0.008	0.03
3	12.36	12.57	0.008	0.03
4	12.51	12.73	0.008	0.03
5	15.07	15.24	0.01	0.03
6	15.49	15.65	0.01	0.02
7	16.91	17.07	0.01	0.02
8	17.66	17.82	0.01	0.02
9	18.35	18.44	0.01	0.02
10	18.66	18.75	0.01	0.02

Table S24: Raw values used for computing the vertical ionization energies (IEs) for the conformation V of the H_2O_2 dimer as determined using the frozen-core EOMIP-CCSD/aug-cc-pVnZ (n = D, T, and Q) approaches. The values are given in hartrees.

**State**	$\mathbf{E}_{AVDZ}$	$\mathbf{E}_{AVTZ}$	$\mathbf{E}_{AVQZ}$
Ground	-302.47050554005915	-302.71800654470354	-302.79435925469670
1	-302.05983067447022	-302.29872460148630	-302.37171941263119
2	-302.04962761010921	-302.28856720091733	-302.36158410631731
3	-302.01676668424841	-302.25647389825218	-302.32963068362818
4	-302.01098233214617	-302.25077783455885	-302.32395485499029
5	-301.91702417456401	-302.15858117265805	-302.23196717500434
6	-301.90154789457944	-302.14338148616667	-302.21684537982446
7	-301.84956565985226	-302.09116291805861	-302.16467820838568
8	-301.82187327446422	-302.06366979370273	-302.13728745108716
9	-301.79629085747547	-302.04065883851393	-302.11447126167457
10	-301.78497533237191	-302.02915112442213	-302.10299688585133

Table S25: Raw values used for computing the vertical ionization energies (IEs) for the conformation V of the H_2O_2 dimer as determined using the EOMIP-CCSD/aug-cc-pCVnZ (n = D and T) approach with all electrons correlated. The values are given in hartrees.

**State**	$\mathbf{E}_{ACVDZ}$	$\mathbf{E}_{ACVTZ}$
Ground	-302.63441084213508	-302.94366408593584
1	-302.22308036106097	-302.52263303211816
2	-302.21288412552275	-302.51249635739265
3	-302.18001764182748	-302.48051732663896
4	-302.17424751114464	-302.47484274519479
5	-302.08026013378242	-302.38286034401699
6	-302.06480045726795	-302.36771115319914
7	-302.01275719269432	-302.31556520437641
8	-301.98506071245339	-302.28813311065949
9	-301.95972902867868	-302.26528368713400
10	-301.94841179913237	-302.25377821152392

Table S26: Raw values used for computing the vertical ionization energies (IEs) for the conformation V of the H_2O_2 dimer as determined using the frozen-core EOMIP-CCSD/aug-cc-pCVnZ (n = D and T) approaches. The values are given in hartrees.

**State**	$\mathbf{E}^{FC}_{ACVDZ}$	$\mathbf{E}^{FC}_{ACVTZ}$
Ground	-302.48198599564341	-302.72976513296965
1	-302.07095738732852	-302.30994964326260
2	-302.06076065126365	-302.29980121430509
3	-302.02787816607872	-302.26773670887491
4	-302.02210516578066	-302.26204723352089
5	-301.92808185643219	-302.16988769658752
6	-301.91261379998599	-302.15470771045625
7	-301.86057777410991	-302.10249441375441
8	-301.83287301746395	-302.07500504335775
9	-301.80752401904533	-302.05218985243755
10	-301.79620851675622	-302.04068263715868

Table S27: Cartesian coordinates of the conformation V of the H_2O_2 dimer as optimized at the CCSD(T)/aug-cc-pVTZ level of theory in the gas-phase. Values are given in Å

Atom	X	Y	Z
O	1.9305662881	0.3711746603	-0.4802760376
O	1.0074752020	-0.1583987755	0.5192123701
O	-1.5852191183	0.9188031698	0.0049163471
O	-1.6043979518	-0.4896840245	-0.3892527536
H	0.4259577730	-0.7013365200	-0.0395302037
H	2.7420042638	-0.0813674263	-0.2134750559
H	-2.2407031319	-0.8345585416	0.2514875865
H	-0.6756833249	0.9753674578	0.3469177471

Table S28: Cartesian coordinates of the cation of conformation V of the H_2O_2 dimer as optimized at the CCSD(T)/aug-cc-pVTZ level of theory in the gas-phase. Values are given in Å

Atom	X	Y	Z
O	1.4659238624	0.0938554948	-0.7822871497
O	1.0515618231	-0.1057009522	0.5991175091
O	-1.2994660468	0.4640120846	0.4962492739
O	-1.8214412873	-0.2715125033	-0.4686444048
H	1.2759466773	-1.0417230262	0.7638600031
H	2.1930866787	0.7302327331	-0.6526269410
H	-2.7611326951	0.0184335408	-0.5114415258
H	-0.1044790123	0.1124026285	0.5557732352

Table S29: Cartesian coordinates of the conformation IV of the H_2O_2 dimer as optimized at the CCSD(T)/aug-cc-pVTZ level of theory in the gas-phase. Values are given in Å

Atom	X	Y	Z
O	-1.4657245878	0.5605462507	0.3178316621
O	-1.6493839791	-0.7299187751	-0.3409863473
O	1.0571171482	-0.5078900133	0.6844844283
O	1.5001431852	0.1077062121	-0.5675563614
H	-0.7496845341	-1.0907377192	-0.2590964349
H	-1.4294059708	1.1430978680	-0.4538523816
H	2.3846094947	0.3967431639	-0.3065524428
H	0.3523292437	0.1204530130	0.9257278777

Table S30: Cartesian coordinates of the conformation VI of the H_2O_2 dimer as optimized at the CCSD(T)/aug-cc-pVTZ level of theory in the gas-phase. Values are given in Å

Atom	X	Y	Z
O	1.6043971631	-0.4896835960	-0.3892534626
O	1.5852179937	0.9188034407	0.0049162319
O	-1.0074755477	-0.1583998782	0.5192137094
O	-1.9305647704	0.3711751810	-0.4802755933
H	0.6756824992	0.9753670486	0.3469185418
H	2.2407033401	-0.8345580165	0.2514859442
H	-2.7420033088	-0.0813671313	-0.2134766953
H	-0.4259573692	-0.7013370484	-0.0395286761

Table S31: Vertical ionization energies (IEs) for the conformation I of the H_2O_2 dimer as determined using the frozen-core EOMIP-CCSD/aug-cc-pVnZ (n = D, T, and Q) approaches along with CBS limit extrapolations. All the values are given in eV.

IE	$\mathbf{E}_{\mathrm{AVDZ}}$	$\mathbf{E}_{\mathrm{AVTZ}}$	$\mathbf{E}_{\mathrm{AVQZ}}$	$\mathbf{E}_{\mathrm{CBS[DT]}}$	$\mathbf{E}_{\mathrm{CBS[TQ]}}$
1	11.31	11.54	11.63	11.62	11.68
2	12.37	12.58	12.66	12.65	12.71
3	15.23	15.38	15.46	15.44	15.51
4	17.25	17.40	17.48	17.46	17.52
5	18.47	18.55	18.62	18.58	18.65
6	20.65	21.37	21.56	21.62	21.68
7	22.30	23.02	23.22	23.27	23.33
8	23.13	23.78	23.96	24.01	24.07
9	23.64	24.28	24.46	24.51	24.57
10	25.68	26.32	26.50	26.54	26.61

Table S32: Core correlation effects for the vertical ionization energies (IEs) of the H_2O_2 molecule as determined using EOMIP-CCSD/aug-cc-pCVnZ (n = D and T) approaches. The values are given in eV.

IE	$\mathbf{E}^{\mathrm{FC}}_{\mathrm{ACVDZ}}$	$\mathbf{E}^{\mathrm{FC}}_{\mathrm{ACVTZ}}$	$\Delta_{\mathrm{CV}}(\mathbf{DZ})$	$\Delta_{\mathrm{CV}}(\mathbf{TZ})$
1	11.32	11.55	0.008	0.03
2	12.38	12.59	0.007	0.03
3	15.24	15.40	0.006	0.02
4	17.26	17.41	0.006	0.02
5	18.48	18.55	0.01	0.02
6	20.68	21.40	0.07	0.15
7	22.32	23.06	0.07	0.15
8	23.16	23.82	0.07	0.15
9	23.67	24.31	0.07	0.14
10	25.71	26.35	0.07	0.14

Table S33: Raw values used for computing the vertical ionization energies (IEs) of the H_2O_2 molecule as determined using the frozen-core EOMIP-CCSD/aug-cc-pVnZ (n = D, T, and Q) approaches. The values are given in hartrees.

**State**	$\mathbf{E}_{AVDZ}$	$\mathbf{E}_{AVTZ}$	$\mathbf{E}_{AVQZ}$
Ground	-151.22990065454110	-151.35396532913879	-151.39234773807414
1	-150.81427626603733	-150.92998110274183	-150.96509367937642
2	-150.77525446746421	-150.89181263130180	-150.92707779808958
3	-150.67010992906970	-150.78860808153848	-150.82413584425242
4	-150.59593700740768	-150.71446686021557	-150.75015076997138
5	-150.55106128178292	-150.67232466334264	-150.70826357540523
6	-150.47100614774018	-150.56872790359060	-150.59991668650173
7	-150.41050568381709	-150.50800156036522	-150.53906537743592
8	-150.37978990261621	-150.48008274793415	-150.51172310906190
9	-150.36131313523722	-150.46175012009999	-150.49345427003499
10	-150.28628417172590	-150.38684511722749	-150.41842681291379

Table S34: Raw values used for computing the vertical ionization energies (IEs) of the H_2O_2 molecule as determined using the EOMIP-CCSD/aug-cc-pCVnZ (n = D and T) approach with all electrons correlated. The values are given in hartrees.

**State**	$\mathbf{E}_{ACVDZ}$	$\mathbf{E}_{ACVTZ}$
Ground	-151.31174332874266	-151.46685486938182
1	-150.89548707751828	-151.04115782124774
2	-150.85646627420334	-151.00311964130785
3	-150.75130171950858	-150.90016318892401
4	-150.67706180890505	-150.82615825132024
5	-150.63245170417144	-150.78421065677850
6	-150.54928181877105	-150.67472710653055
7	-150.48887562670851	-150.61405682843076
8	-150.45796331356863	-150.58628250299265
9	-150.43954063175434	-150.56809219672471
10	-150.36461499819089	-150.49330011197961

Table S35: Raw values used for computing the vertical ionization energies (IEs) of the H_2O_2 molecule as determined using the frozen-core EOMIP-CCSD/aug-cc-pCVnZ (n = D and T) approaches. The values are given in hartrees.

**State**	$\mathbf{E}^{FC}_{ACVDZ}$	$\mathbf{E}^{FC}_{ACVTZ}$
Ground	-151.23561514802498	-151.35994489505543
1	-150.81964736768444	-150.93542709029472
2	-150.78060898743624	-150.89729398356133
3	-150.67540668445218	-150.79414159149388
4	-150.60116132858252	-150.72001119081645
5	-150.55653302569664	-150.67808655544260
6	-150.47570433450588	-150.57335160438481
7	-150.41525668353646	-150.51263896649149
8	-150.38437894274313	-150.48473405952288
9	-150.36591234849251	-150.46643251168007
10	-150.29093538432255	-150.39156430582261

Table S36: Cartesian coordinates of the H_2O_2 molecule as optimized at the CCSD(T)/aug-cc-pVTZ level of theory in the gas-phase. Values are given in Å

Atom	X	Y	Z
O	-0.7220653092	0.1100444099	-0.2657911363
O	0.7221279340	-0.1102225953	-0.2655383223
H	-1.0047137280	-0.6459976584	0.2657586513
H	1.0046511032	0.6461758438	0.2655708074

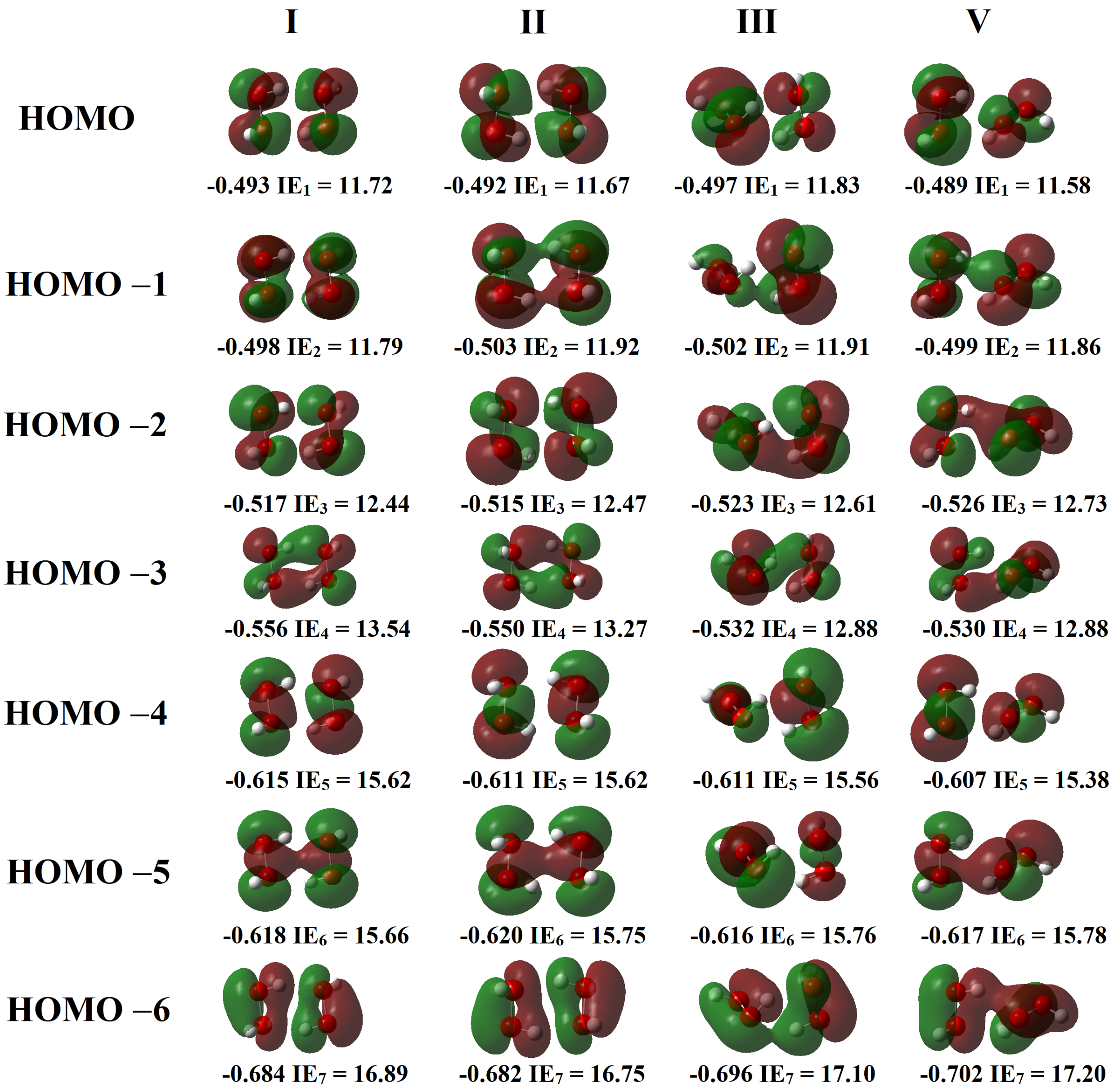

Figure S1: Molecular orbital plots generated at HF/aug-cc-pVTZ for the conformations of the H_2O_2 dimer. The orbital energies are given in hartrees while the corresponding composite IEs are provided in eV.
